# Introduction of Eurasian-Origin Influenza A(H8N4) Virus into North America by Migratory Birds

**DOI:** 10.3201/eid2410.180447

**Published:** 2018-10

**Authors:** Andrew M. Ramey, Andrew B. Reeves, Tyrone Donnelly, Rebecca L. Poulson, David E. Stallknecht

**Affiliations:** US Geological Survey Alaska Science Center, Anchorage, Alaska, USA (A.M. Ramey, A.B. Reeves, T. Donnelly);; University of Georgia, Athens, Georgia, USA (R.L. Poulson, D.E. Stallknecht)

**Keywords:** influenza A(H8N4), viruses, influenza, introduction, North America, virus, wild birds, migratory birds, H8N4, Alaska, United States, USA

## Abstract

We identified a Eurasian-origin influenza A(H8N4) virus in North America by sampling wild birds in western Alaska, USA. Evidence for repeated introductions of influenza A viruses into North America by migratory birds suggests that intercontinental dispersal might not be exceedingly rare and that our understanding of viral establishment is incomplete.

Research of and surveillance for influenza A viruses in wild birds inhabiting western Alaska have consistently provided support for the exchange of viruses between East Asia and North America via Beringia (*1*,*2*). Sampling of wild birds inhabiting Izembek National Wildlife Refuge (NWR) and surrounding areas in Alaska (≈55°N, 163°W) conducted during 2011–2015 has been used in recent research to identify the dispersal of influenza A(H9N2) viruses among China, South Korea, and Alaska (*3*); provide inference about the evolutionary pathways of economically important foreign-origin poultry pathogens introduced into North America (*4*); and identify sampling efficiencies for optimizing the detection of evidence for intercontinental virus exchange (*5*).

During September–October 2016, we collected 541 combined oral-pharyngeal and cloacal swab samples from hunter-harvested waterfowl (*Anseriformes* spp.) and 401 environmental fecal samples from monospecific flocks of either emperor geese (*Chen canagica*) or glaucous-winged gulls (*Larus glaucescens*) within and around Izembek NWR. Samples were deposited into viral transport media, placed in dry shippers charged with liquid nitrogen within 24 h, shipped, and stored frozen at −80°C before laboratory analysis. We screened samples for the influenza A virus matrix gene and subjected them to virus isolation; resultant isolates were genomically sequenced in accordance with previously reported methods (*5*). A total of 116 samples tested positive for the matrix gene, and 38 isolates were recovered of the following combined subtypes: H1N2, H3N2, H3N2/N6 (mixed infection), H3N8, H4N6, H5N2, H6N2, H7N3, H8N4, and H12N2. We selected the single H8N4 isolate, A/northern pintail/Alaska/UGAI16-3997/2016(H8N4) (GenBank accession nos. MG976689–96), for genomic characterization as part of this investigation.

We queried sequence information for the complete coding region of each gene segment of A/northern pintail/Alaska/UGAI16-3997/2016(H8N4) against the GenBank database to identify strains sharing >99% nt identity. We then reconstructed maximum-likelihood phylogenetic trees for each gene segment in MEGA 7.0.21 (https://www.megasoftware.net/) by incorporating sequence information for representative reference sequences from avian-origin influenza A virus isolates from Eurasia and North America using the general time-reversible plus invariant sites (G+I) model with 1,000 bootstrap replications.

Gene segments for A/northern pintail/Alaska/UGAI16-3997/2016(H8N4), isolated from a sample collected from a hunter-harvested duck on September 6, 2016, shared >99% nt identity to those of >1 isolates recovered from wild and domestic birds sampled in East Asia during 2006–2016 (Technical Appendix Table). This isolate also shared >99% nt identity with 1–4 isolates recovered from wild bird samples collected at Izembek NWR during 2012–2015 at the polymerase acidic and polymerase basic 2 gene segments (Technical Appendix Table). A/northern pintail/Alaska/UGAI16–3997/2016(H8N4) did not, however, share >99% nt identity at all 8 gene segments with any other influenza A virus isolate for which genomic information was available, indicating that this H8N4 isolate might represent a previously unidentified or unreported genome constellation (Technical Appendix Table).

Phylogenetic analyses strongly supported structuring of tree topologies into major clades by continental affiliation of reference sequences (bootstrap values >99; Technical Appendix Figure). Sequence information for all 8 gene segments of A/northern pintail/Alaska/UGAI16-3997/2016(H8N4) clustered within clades composed of reference sequences for influenza A viruses originating from samples collected in Eurasia (Figure; Technical Appendix Figure). Therefore, phylogenetic analyses provided support for Eurasian ancestry of this genomic constellation. We inferred our results to provide evidence for the introduction of this foreign-origin H8N4 virus into North America by migratory birds given previous support for intercontinental viral dispersal derived through genetic characterization of avian influenza A viruses originating from western Alaska (*1*–*3*,*5*), the intercontinental migratory tendencies of northern pintails (*6*,*7*) and other species inhabiting Izembek NWR at the time of sampling (*8*), the paucity of domestic poultry in this region, and the proximity of Izembek NWR to East Asia.

**Figure:**
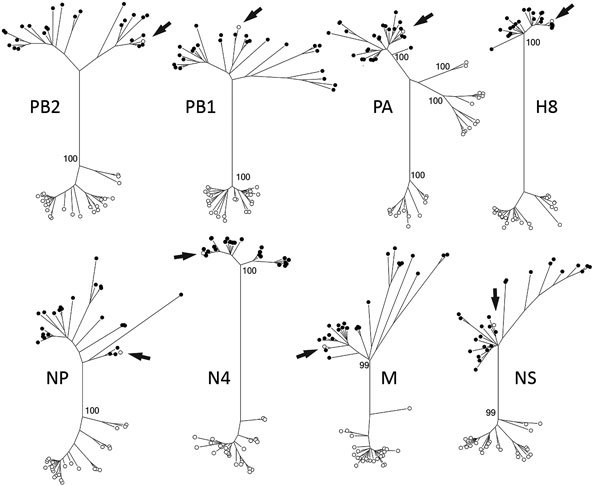
Maximum-likelihood phylogenetic trees showing inferred relationships among nucleotide sequences for the complete coding regions of gene segments for influenza A virus strain A/northern pintail/Alaska/UGAI16–3997/2016(H8N4) (white circle indicated with an arrow) and reference sequences from viruses isolated from birds in Eurasia (black circles) and North America (white circles). Bootstrap support values for continentally affiliated clades are shown. Phylogenetic trees with complete strain names as tip labels are provided in the online Technical Appendix Figure (https://wwwnc.cdc.gov/EID/article/24/10/18-0447-Techap1.pdf). H, hemagglutinin; M, matrix; N, neuraminidase; NP, nucleoprotein; NS, nonstructural; PA, polymerase acidic; PB, polymerase basic.

During 2010–2016, research and surveillance for influenza A viruses in wild birds inhabiting North America have provided evidence for the intercontinental dispersal of the following 4 viral genome constellations between Eurasia and North America: H16N3 (*9*), H9N2 (*3*), highly pathogenic clade 2.3.4.4 H5N8 (*10*), and H8N4 (this study). Four reports of independent purported intercontinental dispersal events for influenza A viruses via migratory birds during 7 years of sampling do not disprove the paradigm of restricted viral dispersal between Eurasia and North America. However, repeated detections of these viruses crossing the Bering Strait (*3*,*10*; this study) suggest that viral dispersal between East Asia and North America might not be exceedingly rare. Thus, a lack of selective advantage for comparatively rare foreign-origin influenza A viruses, purifying selection for endemic viruses, or both might be important mechanisms regulating the establishment of these viruses within the wild bird reservoir. Therefore, additional research directed toward understanding selection pressures regulating the establishment of these viruses might provide useful inference for informing surveillance and response activities for economically costly or potentially pandemic foreign-origin viruses in wild birds inhabiting North America.

Technical Appendix Viral isolates sharing ≥99% nt identity at >1 gene segments with A/northern pintail/Alaska/UGAI16–3997/2016(H8N4), March 12, 2018; unrooted maximum-likelihood phylogenetic trees for the complete coding regions of the gene segments for influenza A virus strain A/northern pintail/Alaska/UGAI16–3997/2016(H8N4).
