# Supplementary material for: Introduction of Eurasian-Origin Influenza A(H8N4) Virus into North America by Migratory Birds
Source: Emerg Infect Dis. 2018 Oct;24(10):1950–3. doi: 10.3201/eid2410.180447 (PMC6154152; doi:10.3201/eid2410.180447)
Supplement: Technical Appendix — Viral isolates sharing ≥99% nt identity at >1 gene segments with A/northern pintail/Alaska/UGAI16–3997/2016(H8N4), March 12, 2018; unrooted maximum-likelihood phylogenetic trees for the complete coding regions of the gene segments for influenza A virus strain A/northern pintail/Alaska/UGAI16–3997/2016(H8N4). [file 18-0447-Techapp-s1.pdf]

Article DOI: https://doi.org/10.3201/eid2410.180447

Introduction of Eurasian-origin Influenza A(H8N4) Virus into North America by Migratory Birds

Technical Appendix

Technical Appendix Table. Virus isolates sharing >99% nt identity at >1 gene segments with A/northern pintail/Alaska/UGAI16–3997/2016(H8N4) as identified on National Center for Biotechnology Information GenBank database on March 12, 2018

Segment	GenBank accession no.	Strain name	Shared nucleotide identity
PB2	KY131326.1	A/northern pintail/Alaska/UGAI15-6994/2015(H6N1)	99%
PB2	KY131357.1	A/northern pintail/Alaska/UGAI15-7300/2015(H6N1)	99%
PB2	KX949521.1	A/northern pintail/Alaska/UGAI15-7403/2015(H3N8)	99%
PB2	LC053481.1	A/duck/Vietnam/LBM798/2014(H3N6)	99%
PB2	KU881717.1	A/Anseriformes/Anhui/L259/2014(H1N1)	99%
PB2	LC148827.1	A/duck/Hokkaido/W9/2015(H1N1)	99%
PB2	LC339728.1	A/duck/Hokkaido/10/2015(H3N6)	99%
PB2	KU881701.1	A/Anseriformes/Anhui/L25/2014(H1N1)	99%
PB2	KT717295.1	A/Anser fabalis/China/Anhui/L221/2014(H6N1)	99%
PB2	KY415872.1	A/duck/Hubei/ZYSYG15/2015(H6N2)	99%
PB2	KY415871.1	A/duck/Hubei/ZYSYG14/2015(H6N2)	99%
PB2	KY415870.1	A/duck/Hubei/ZYSYG1/2015(H6N2)	99%
PB2	KU143586.1	A/duck/Wuhan/WHYF05/2014(H9N2)	99%
PB2	KU881669.1	A/Anseriformes/Anhui/S3/2014(H1N1)	99%
PB2	KY415879.1	A/duck/Hubei/ZYSYF8/2015(H6N6)	99%
PB2	KX121185.1	A/bean goose/Hubei/SZY200/2016(H11N9)	99%
PB1	LC339617.1	A/duck/Hokkaido/W165/2015(H11N6)	99%
PA	LC371817.1	A/duck/Hokkaido/207/2014(H8N2)	99%
PA	KY130810.1	A/glaucous-winged gull/Alaska/473/2012(H3N8)	99%
PA	LC042057.1	A/duck/Hokkaido/W280/2014(H5N3)	99%
PA	LC042050.1	A/duck/Hokkaido/W240/2014(H5N3)	99%
PA	LC339634.1	A/duck/Hokkaido/W19/2013(H7N2)	99%
PA	KU160978.1	A/duck/Hunan/S1012/2009(H4N6)	99%
PA	KY971098.1	A/pigeon/Zhejiang/1120087/2014(H1N2)	99%
PA	KY971097.1	A/goose/Zhejiang/1120085/2014(H1N2)	99%
PA	KY971096.1	A/goose/Zhejiang/1120084/2014(H1N2)	99%
PA	KY971095.1	A/goose/Zhejiang/1120083/2014(H1N2)	99%
PA	KY971094.1	A/goose/Zhejiang/1120078/2014(H1N2)	99%
PA	KY971092.1	A/goose/Zhejiang/1120074/2014(H1N2)	99%
PA	LC339530.1	A/duck/Hokkaido/201/2014(H1N1)	99%
PA	KY971093.1	A/goose/Zhejiang/1120076/2014(H1N2)	99%
PA	KY971091.1	A/goose/Zhejiang/1120069/2014(H1N2)	99%
PA	JX454750.1	A/wild duck/Korea/SH5-26/2008(H4N6)	99%
PA	GU086282.1	A/duck/Korea/A93/2008(H5N2)	99%
PA	GQ325638.1	A/environment/Dongting Lake/Hunan/3-9/2007(H10N8)	99%
PA	GQ290468.1	A/environment/Dongting Lake/Hunan/3-9/2007(H10N8)	99%
PA	KU160938.1	A/duck/Henan/S1091/2010(H4N6)	99%
PA	JQ041395.1	A/duck/Hebei/0908/2009(H5N2)	99%
PA	JX454742.1	A/wild duck/Korea/PSC6-1/2009(H4N6)	99%
PA	HM745397.1	A/duck/Jiangxi/k0701/2009(H11N2)	99%
PA	KC899731.1	A/wild duck/SH38-64/2010(H2N8)	99%
PA	CY098237.1	A/aquatic bird/Korea/CN9/2009(H6N8)	99%
PA	GQ325654.1	A/environment/Dongting Lake/Hunan/3-9/2007(H10N8)	99%
PA	KP862040.1	A/duck/Hunan/S4280/2009(H10N8)	99%
PA	KC899729.1	A/wild duck/SH38-56/2010(H2N8)	99%
PA	KF260286.1	A/duck/Yunnan/87/2007(H7N6)	99%
PA	KF260249.1	A/duck/Jiangxi/21669/2009(H7N7)	99%
PA	KX297824.1	A/environment/Korea/W148/2006(H7N7)	99%
PA	KX297823.1	A/environment/Korea/W143/2006(H7N7)	99%
PA	KU160930.1	A/duck/Guizhou/S1167/2010(H4N6)	99%

Segment	GenBank accession no.	Strain name	Shared nucleotide identity
PA	KF260254.1	A/chicken/Jiangxi/2369/2010(H7N7)	99%
PA	JX454718.1	A/duck/Korea/DY104/2007(H4N6)	99%
PA	CY049803.1	A/gadwall/Altai/1326/2007(H3N8)	99%
PA	KU921397.1	A/duck/Shanghai/602/2009(H10N8)	99%
PA	KX297827.1	A/environment/Korea/W169/2007(H7N7)	99%
PA	KP287965.1	A/duck/Jiangxi/26141/2009(H10N7)	99%
PA	KF260251.1	A/duck/Jiangxi/23008/2009(H7N7)	99%
HA	LC339587.1	A/duck/Hokkaido/95/2014(H8N4)	99%
NP	LC339692.1	A/duck/Hokkaido/WZ1/2014(H11N2)	99%
NP	LC371795.1	A/duck/Hokkaido/166/2014(H5N2)	99%
NP	LC042066.1	A/duck/Hokkaido/WZ1/2014(H11N2)	99%
NP	LC339612.1	A/duck/Hokkaido/W150/2014(H4N6)	99%
NP	LC339596.1	A/duck/Hokkaido/W118/2014(H4N6)	99%
NP	CY079214.1	A/avian/Japan/8KI0195/2008(H6N8)	99%
NP	AB530993.1	A/mallard/Hokkaido/24/2009(H5N1)	99%
NP	KF259795.1	A/common teal/Hong Kong/MPK630/2009(H10N9)	99%
NP	KF259796.1	A/eurasian wigeon/Hong Kong/MPK653/2009(H11N9)	99%
NP	KF259784.1	A/eurasian wigeon/Hong Kong/MPK655/2009(H11N9)	99%
NP	JX570854.1	A/canvasback/Xianghai/428/2011(H5N2)	99%
NP	LC121485.1	A/duck/Hokkaido/W90/2007(H10N7)	99%
NP	JX570870.1	A/green-winged teal/Xianghai/430/2011(H5N2)	99%
NP	CY079238.1	A/avian/Japan/8KI0150/2008(H3N8)	99%
NP	AB546150.1	A/pintail/Aomori/422/2007(H1N1)	99%
NA	LC339589.1	A/duck/Hokkaido/95/2014(H8N4)	99%
N4	LC339565.1	A/duck/Hokkaido/238/2014(H8N4)	99%
N4	LC339557.1	A/duck/Hokkaido/222/2014(H8N4)	99%
N4	LC339549.1	A/duck/Hokkaido/221/2014(H8N4)	99%
N4	LC339541.1	A/duck/Hokkaido/220/2014(H8N4)	99%
N4	KU143355.1	A/duck/Wenzhou/YJYF78/2015(H1N4)	99%
N4	JN817552.1	A/wild bird/Korea/A02/2011(H10N4)	99%
M	KU881675.1	A/Anseriformes/Anhui/S3/2014(H1N1)	99%
M	KU881691.1	A/Anseriformes/Anhui/S107/2014(H1N1)	99%
M	KU881707.1	A/Anseriformes/Anhui/L25/2014(H1N1)	99%
M	KU881723.1	A/Anseriformes/Anhui/L259/2014(H1N1)	99%
M	KX066874.1	A/wild bird/Korea/SK14/2014(H1N1)	99%
M	KT717285.1	A/Anser fabalis/China/Anhui/L144/2014(H6N1)	99%
M	KT717301.1	A/Anser fabalis/China/Anhui/L221/2014(H6N1)	99%
M	KU143301.1	A/wild bird/Wuhan/CDHN09/2015(H6N2)	99%
M	KU143302.1	A/wild bird/Wuhan/CDHN15/2015(H6N2)	99%
M	LC053487.1	A/duck/Vietnam/LBM798/2014(H3N6)	99%
M	LC121311.1	A/duck/Mongolia/179/2015(H3N8)	99%
M	LC121303.1	A/duck/Mongolia/173/2015(H3N8)	99%
M	KU160854.1	A/duck/Chongqing/S2086/2012(H4N8)	99%
M	KJ907490.1	A/black-necked crane/Zhaotong/ZT-12/2013(H1N2)	99%
M	MF147259.1	A/mallard duck/Netherlands/8/2014(H7N5)	99%
M	KX978943.1	A/mallard duck/Netherlands/2/2015(H7N7)	99%
M	KX977880.1	A/mallard duck/Netherlands/3/2015(H7N7)	99%
M	LC121439.1	A/duck/Mongolia/709/2015(H10N7)	99%
M	LC121423.1	A/duck/Mongolia/626/2015(H10N7)	99%
M	LC121383.1	A/duck/Mongolia/499/2015(H10N7)	99%
M	KF462313.1	A/European teal/Chany/63/2011(H3N8)	99%
M	KY635804.1	A/duck/Bangladesh/24705/2015(H7N1)	99%
M	KY635683.1	A/duck/Bangladesh/24704/2015(H15N9)	99%
M	KY402068.1	A/hooded crane/Korea/1176/2016(H1N1)	99%
M	AB981453.1	A/muscovy duck/Quang Ninh/131/2013(H3N8)	99%
M	CY146551.1	A/duck/Hunan/S11090/2012(H4N6)	99%
NS	KU881716.1	A/Anseriformes/Anhui/L167/2014(H1N1)	99%
NS	KT717222.1	A/Anser fabalis/China/Anhui/S39/2014(H6N2)	99%
NS	KT717254.1	A/Anser fabalis/China/Anhui/S148/2014(H6N2)	99%
NS	KU143433.1	A/chicken/Wuhan/WHJF/2014(H5N2)	99%
NS	KT717230.1	A/Anser fabalis/China/Anhui/S45/2014(H6N2)	99%
NS	KT717246.1	A/Anser fabalis/China/Anhui/S104/2014(H6N2)	99%
NS	KU160967.1	A/duck/Hubei/S2213/2012(H4N2)	99%
NS	KJ526011.1	A/duck/Thailand/CU-11836C/2011(H1N3)	99%
NS	KC609961.1	A/mallard/Korea/NHG187/2008(H7N7)	99%
NS	MG367026.1	A/grey heron/Korea/VI1511105/2015(H3N8)	99%
NS	MG367025.1	A/mallard duck/Korea/VI1511095/2015(H4N6)	99%
NS	MG367024.1	A/mallard duck/Korea/VI1511090/2015(H4N6)	99%
NS	MG367023.1	A/great egret/Korea/VI1511065/2015(H4N6)	99%

Segment	GenBank accession no.	Strain name	Shared nucleotide identity
NS	MG367022.1	A/mallard duck/Korea/VI160646/2016(H6N5)	99%
NS	MG367021.1	A/mallard duck/Korea/VI160383/2016(H6N2)	99%
NS	MG367020.1	A/mallard duck/Korea/VI160266/2016(H10N1)	99%
NS	MG367019.1	A/falcata teal/Korea/VI160246/2016(H11N9)	99%
NS	MG367018.1	A/green-winged teal/Korea/VI160181/2016(H10N1)	99%
NS	MG367017.1	A/green-winged teal/Korea/VI160151/2016(H11N9)	99%
NS	MG367016.1	A/mallard duck/Korea/VI154461/2015(H4N6)	99%
NS	MG367015.1	A/mallard duck/Korea/VI152573/2015(H11N9)	99%
NS	MG367014.1	A/mallard duck/Korea/VI152538/2015(H5N3)	99%
NS	MG367013.1	A/mallard duck/Korea/VI152533/2015(H5N3)	99%
NS	MG367012.1	A/mallard duck/Korea/VI147540/2014(H11N9)	99%
NS	MG367011.1	A/mallard duck/Korea/VI147532/2014(H11N9)	99%
NS	MG367010.1	A/mallard duck/Korea/VI146993/2014(H3N8)	99%
NS	MG367009.1	A/mallard duck/Korea/VI142218/2014(H3N8)	99%
NS	MG367008.1	A/mallard duck/Korea/VI141432/2014(H3N8)	99%
NS	MG367007.1	A/mallard duck/Korea/VI141407/2014(H7N7)	99%
NS	MG367006.1	A/hooded crane/Korea/VI160792/2016(H4N8)	99%
NS	MG367005.1	A/mallard duck/Korea/VI150161/2015(H7N6)	99%
NS	MG367004.1	A/mallard duck/Korea/VI150051/2015(H7N1)	99%
NS	MG367003.1	A/mallard duck/Korea/CBU143781/2014(H1N8)	99%
NS	MG367002.1	A/mallard duck/Korea/CBU143751/2014(H4N1)	99%
NS	MG367001.1	A/mallard duck/Korea/CBU143501/2014(H4N1)	99%
NS	MG367000.1	A/mallard duck/Korea/CBU143491/2014(H4N6)	99%
NS	MG366999.1	A/mallard duck/Korea/CBU142416/2014(H3N6)	99%
NS	MG366998.1	A/hooded crane/Korea/VI1513992/2015(H3N8)	99%
NS	MG366997.1	A/Northern pintail/Korea/VI1511874/2015(H3N8)	99%
NS	MG366996.1	A/mallard duck/Korea/VI1511070/2015(H10N5)	99%
NS	MG366995.1	A/falcata teal/Korea/VI160216/2016(H2N5)	99%
NS	MG366994.1	A/mallard duck/Korea/VI146916/2014(H7N4)	99%
NS	MG366993.1	A/mallard duck/Korea/VI146688/2014(H6N8)	99%
NS	MG366992.1	A/mallard duck/Korea/VI146668/2014(H6N8)	99%
NS	MG366991.1	A/mallard duck/Korea/VI145825/2014(H7N7)	99%
NS	MG366990.1	A/mallard duck/Korea/VI142063/2014(H7N7)	99%
NS	MG366989.1	A/mallard duck/Korea/VI141417/2014(H3N8)	99%
NS	MG366988.1	A/hooded crane/Korea/VI160196/2016(H1N1)	99%
NS	MG366987.1	A/mallard duck/Korea/CBU142426/2014(H4N2)	99%
NS	MG366985.1	A/mallard duck/Korea/VI141467/2014(H3N8)	99%
NS	LC339735.1	A/duck/Hokkaido/10/2015(H3N6)	99%
NS	KC609960.1	A/Northern shoveler/Korea/SD175/2008(H7N3)	99%
NS	KC899765.1	A/wild duck/SH17-34/2008(H2N3)	99%
NS	KF260044.1	A/duck/Yunnan/2908/2009(H11N9)	99%
NS	JX454749.1	A/wild duck/Korea/SH5-26/2008(H4N6)	99%
NS	LC371790.1	A/duck/Hokkaido/162/2013(H2N1)	99%
NS	MG366986.1	A/mallard duck/Korea/VI141412/2014(H1N1)	99%
NS	KX162596.1	A/duck/Shanghai/408-1/2009(H4N6)	99%
NS	KF357802.1	A/duck/Zhejiang/4613/2013(H3N2)	99%
NS	KJ526003.1	A/duck/Thailand/CU-12677T/2012(H11N6)	99%
NS	JX570845.1	A/spot-billed duck/Xianghai/427/2011(H5N2)	99%
NS	JN852799.1	A/chicken/Korea/KNUSWR09/2009(H9N2)	99%
NS	JN852807.1	A/chicken/Korea/KNUWSJ09/2009(H9N2)	99%
NS	JN087364.1	A/environment/Korea/SH11-10/2009(H3N8)	99%
NS	HQ897969.1	A/mallard/Korea/KNU YP09/2009(H1N1)	99%
NS	GU086262.1	A/duck/Korea/A14/2008(H5N2)	99%
NS	KY785842.1	A/chicken/Korea/C47/2009(H9N2)	99%
NS	KX162597.1	A/duck/Shanghai/420-2/2009(H4N6)	99%
NS	KX297819.1	A/environment/Korea/W478/2014(H7N7)	99%
NS	KX121192.1	A/bean goose/Hubei/SZY200/2016(H11N9)	99%
NS	KJ525995.1	A/duck/Thailand/CU-12679C/2012(H4N6)	99%
NS	KJ525991.1	A/duck/Thailand/CU-12659T/2012(H4N6)	99%
NS	JN852791.1	A/chicken/Korea/KNUGJ09/2009(H9N2)	99%
NS	CY079247.1	A/avian/Japan/8KI0135/2008(H6N5)	99%
NS	GU086258.1	A/chicken/Korea/A170/2009(H9N2)	99%
NS	LC367414.1	A/duck/Mongolia/53/2011(H3N8)	99%
NS	LC367406.1	A/duck/Mongolia/50/2010(H10N8)	99%
NS	LC367398.1	A/duck/Mongolia/496/2010(H3N3)	99%
NS	LC367374.1	A/duck/Mongolia/46/2010(H10N8)	99%
NS	KT266946.1	A/duck/Guangxi/113/2012(H6N8)	99%
NS	KX297813.1	A/environment/Korea/W178/2007(H7N7)	99%
NS	KX297811.1	A/environment/Korea/W156/2006(H7N7)	99%

Segment	GenBank accession no.	Strain name	Shared nucleotide identity
NS	LC042061.1	A/duck/Hokkaido/W280/2014(H5N3)	99%
NS	LC042054.1	A/duck/Hokkaido/W240/2014(H5N3)	99%
NS	KF260030.1	A/northern shoveler/Hong Kong/MPC657/2006(H10N9)	99%
NS	KC693602.1	A/duck/Taiwan/DV1846/2010(H3N8)	99%
NS	JN244259.1	A/duck/Korea/A349/2009(H7N2)	99%
NS	JN852783.1	A/duck/Korea/KNUDPJ09/2009(H9N2)	99%
NS	AB593435.1	A/duck/Vietnam/G119/2006(H3N8)	99%
NS	FJ750868.1	A/mallard/Korea/GH170/2007(H7N7)	99%
NS	GQ414964.1	A/spot-billed duck/Korea/528/2008(H6N8)	99%
NS	LC339527.1	A/duck/Hokkaido/20/2015(H3N8)	99%
NS	LC332535.1	A/duck/Hokkaido/17/2015(H3N8)	99%
NS	LC332527.1	A/duck/Hokkaido/15/2015(H3N8)	99%
NS	LC332519.1	A/duck/Hokkaido/14/2015(H3N8)	99%
NS	LC332511.1	A/duck/Hokkaido/13/2015(H3N8)	99%
NS	LC332503.1	A/duck/Hokkaido/12/2015(H3N8)	99%
NS	GU086257.1	A/chicken/Korea/A146/2009(H9N2)	99%
NS	LC367502.1	A/duck/Mongolia/675/2010(H1N1)	99%
NS	LC367390.1	A/duck/Mongolia/493/2010(H10N8)	99%
NS	LC349403.1	A/duck/Mongolia/371/2010(H10N8)	99%
NS	LC349395.1	A/duck/Mongolia/340/2011(H4N8)	99%
NS	KY785818.1	A/chicken/Korea/C36/2009(H9N2)	99%
NS	KX297810.1	A/environment/Korea/W152/2006(H7N7)	99%
NS	KU158918.1	A/chicken/Nanjing/B854-2/2011(H3N8)	99%
NS	KU158917.1	A/duck/Nanjing/A1591-1/2010(H3N8)	99%
NS	KT318510.1	A/duck/Thailand/CU-11671C/2011(H3N8)	99%
NS	KF886485.1	A/wild bird/Jilin/SJ95/2012(H9N2)	99%
NS	LC000592.1	A/muscovy duck/Vietnam/LBM687/2014(H4N6)	99%
NS	KF260026.1	A/common teal/Hong Kong/MPL1075/2011(H11N9)	99%
NS	KF013922.1	A/duck/Guangxi/GXd-1/2011(H1N2)	99%
NS	KC876695.1	A/wild goose/Dongting/C1037/2011(H12N8)	99%
NS	JX273028.1	A/duck/Korea/J31/2009(H7N2)	99%
NS	AB593467.1	A/duck/Vietnam/G30/2008(H11N9)	99%
NS	GQ414969.1	A/spot-billed duck/Korea/546/2008(H6N1)	99%
NS	GQ414968.1	A/spot-billed duck/Korea/536/2008(H6N1)	99%
NS	GQ414965.1	A/mallard/Korea/L08-8/2008(H6N1)	99%
NS	GQ414963.1	A/spot-billed duck/Korea/527/2008(H6N1)	99%
NS	GQ414970.1	A/spot-billed duck/Korea/537/2008(H6N1)	99%
NS	LC367422.1	A/duck/Mongolia/565/2011(H8N4)	99%
NS	LC349339.1	A/duck/Mongolia/258/2011(H8N4)	99%
NS	LC339815.1	A/duck/Mongolia/121/2011(H10N7)	99%
NS	MG021169.1	A/duck/Jiangshu/YZ916/2016(H3N2)	99%
NS	KY785878.1	A/duck/Korea/D30/2009(H9N2)	99%
NS	KY785810.1	A/chicken/Korea/C31/2009(H9N2)	99%
NS	KU921413.1	A/duck/Fujian/1761/2010(H10N3)	99%
NS	KX867861.1	A/wild bird/Jiangxi/P419/2016(H6N8)	99%
NS	KX028846.1	A/duck/Zhejiang/727D26/2013(H11N3)	99%
NS	KX028845.1	A/duck/Zhejiang/727D25/2013(H11N3)	99%
NS	KX028844.1	A/duck/Zhejiang/727D22/2013(H11N3)	99%
NS	KX028842.1	A/duck/Zhejiang/727D7/2013(H11N3)	99%
NS	KX028841.1	A/duck/Zhejiang/727D2/2013(H11N3)	99%
NS	KU161015.1	A/duck/Jiangxi/S21046/2012(H4N2)	99%
NS	KR265548.1	A/duck/Japan/11OG1032/2011(H5N2)	99%
NS	KR265556.1	A/duck/Japan/11OG1038/2011(H5N2)	99%
NS	KR265564.1	A/duck/Japan/11OG1083/2011(H5N2)	99%
NS	KR265572.1	A/duck/Japan/11OG1084/2011(H5N2)	99%
NS	KP416974.1	A/duck/Jiangxi/5416/2014(mixed)	99%
NS	KP285481.1	A/duck/Jiangxi/15846/2013(H10N3)	99%
NS	KC871461.1	A/mallard/Mongolia/1581/2010(H3N8)	99%
NS	KF260070.1	A/wild waterfowl/Hong Kong/MPM3375/2011(H7N6)	99%
NS	KF260001.1	A/duck/Jiangxi/13291/2009(H7N3)	99%
NS	CY146576.1	A/duck/Hunan/S11313/2012(H4N2)	99%
NS	KF013930.1	A/duck/Guangxi/GXd-4/2011(H1N2)	99%
NS	AB546184.1	A/pintail/Aomori/1130/2008(H1N3)	99%
NS	CY060339.1	A/mallard/Sweden/58/2003(H11N1)	99%
NS	FJ802405.1	A/duck/Thailand/AY-354/2008(H3N2)	99%
NS	EU580556.1	A/Anas querquedula/Astrakhan/3091/2002(H4N8)	99%
NS	EU158147.1	A/duck/Jiangxi/1742/03(H7N7)	99%
NS	GU086259.1	A/duck/Korea/A174/2009(H9N2)	99%

*M, matrix; N, neuraminidase; NP, nucleoprotein; NS, nonstructural; PA, polymerase acidic; PB, polymerase basic.

A

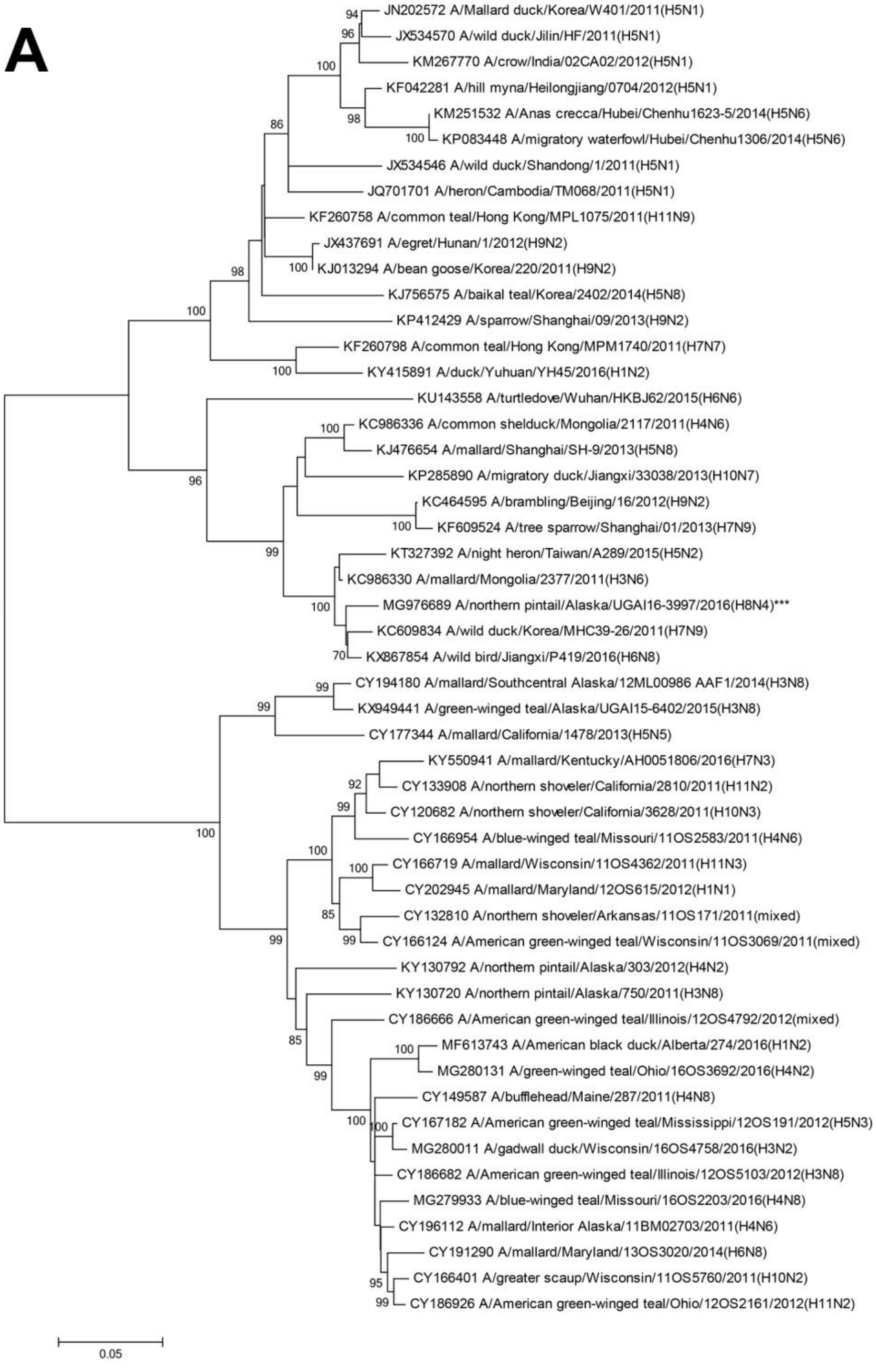
JN202572 A/Mallard duck/Korea/W401/2011(H5N1)
JX534570 A/wild duck/Jilin/HF/2011(H5N1)
KM267770 A/crow/India/02CA02/2012(H5N1)
KF042281 A/hill myna/Heilongjiang/0704/2012(H5N1)
KM251532 A/Anas crecca/Hubei/Chenhu1623-5/2014(H5N6)
KP083448 A/migratory waterfowl/Hubei/Chenhu1306/2014(H5N6)
JX534546 A/wild duck/Shandong/1/2011(H5N1)
JQ701701 A/heron/Cambodia/TM068/2011(H5N1)
KF260758 A/common teal/Hong Kong/MPL1075/2011(H11N9)
JX437691 A/egret/Hunan/1/2012(H9N2)
KJ013294 A/bean goose/Korea/220/2011(H9N2)
KJ756575 A/baikal teal/Korea/2402/2014(H5N8)
KP412429 A/sparrow/Shanghai/09/2013(H9N2)
KF260798 A/common teal/Hong Kong/MPM1740/2011(H7N7)
KY415891 A/duck/Yuhuan/YH45/2016(H1N2)
KU143558 A/turtledove/Wuhan/HKBJ62/2015(H6N6)
KC986336 A/common shelduck/Mongolia/2117/2011(H4N6)
KJ476654 A/mallard/Shanghai/SH-9/2013(H5N8)
KP285890 A/migratory duck/Jiangxi/33038/2013(H10N7)
KC464595 A/brambling/Beijing/16/2012(H9N2)
KF609524 A/tree sparrow/Shanghai/01/2013(H7N9)
KT327392 A/night heron/Taiwan/A289/2015(H5N2)
KC986330 A/mallard/Mongolia/2377/2011(H3N6)
MG976689 A/northern pintail/Alaska/UGAI16-3997/2016(H8N4)***
KC609834 A/wild duck/Korea/MHC39-26/2011(H7N9)
KX867854 A/wild bird/Jiangxi/P419/2016(H6N8)
CY194180 A/mallard/Southcentral Alaska/12ML00986 AAF1/2014(H3N8)
KX949441 A/green-winged teal/Alaska/UGAI15-6402/2015(H3N8)
CY177344 A/mallard/California/1478/2013(H5N5)
KY550941 A/mallard/Kentucky/AH0051806/2016(H7N3)
CY133908 A/northern shoveler/California/2810/2011(H11N2)
CY120682 A/northern shoveler/California/3628/2011(H10N3)
CY166954 A/blue-winged teal/Missouri/11OS2583/2011(H4N6)
CY166719 A/mallard/Wisconsin/11OS4362/2011(H11N3)
CY202945 A/mallard/Maryland/12OS615/2012(H1N1)
CY132810 A/northern shoveler/Arkansas/11OS171/2011(mixed)
CY166124 A/American green-winged teal/Wisconsin/11OS3069/2011(mixed)
KY130792 A/northern pintail/Alaska/303/2012(H4N2)
KY130720 A/northern pintail/Alaska/750/2011(H3N8)
CY186666 A/American green-winged teal/Illinois/12OS4792/2012(mixed)
MF613743 A/American black duck/Alberta/274/2016(H1N2)
MG280131 A/green-winged teal/Ohio/16OS3692/2016(H4N2)
CY149587 A/bufflehead/Maine/287/2011(H4N8)
CY167182 A/American green-winged teal/Mississippi/12OS191/2012(H5N3)
MG280011 A/gadwall duck/Wisconsin/16OS4758/2016(H3N2)
CY186682 A/American green-winged teal/Illinois/12OS5103/2012(H3N8)
MG279933 A/blue-winged teal/Missouri/16OS2203/2016(H4N8)
CY196112 A/mallard/Interior Alaska/11BM02703/2011(H4N6)
CY191290 A/mallard/Maryland/13OS3020/2014(H6N8)
CY166401 A/greater scaup/Wisconsin/11OS5760/2011(H10N2)
CY186926 A/American green-winged teal/Ohio/12OS2161/2012(H11N2)
0.05

B

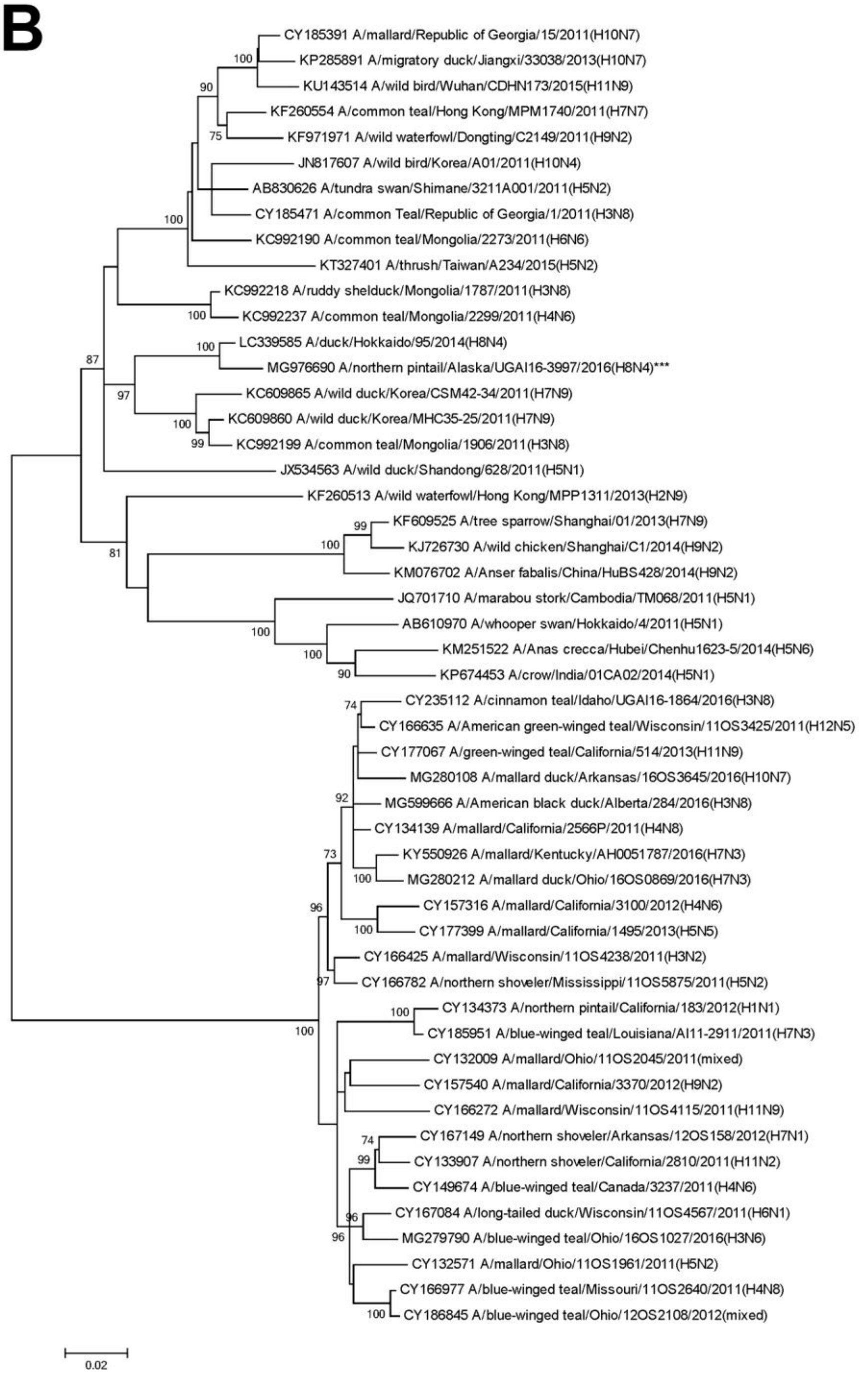
CY185391 A/mallard/Republic of Georgia/15/2011(H10N7)
KP285891 A/migratory duck/Jiangxi/33038/2013(H10N7)
KU143514 A/wild bird/Wuhan/CDHN173/2015(H11N9)
KF260554 A/common teal/Hong Kong/MPM1740/2011(H7N7)
KF971971 A/wild waterfowl/Dongting/C2149/2011(H9N2)
JN817607 A/wild bird/Korea/A01/2011(H10N4)
AB830626 A/tundra swan/Shimane/3211A001/2011(H5N2)
CY185471 A/common Teal/Republic of Georgia/1/2011(H3N8)
KC992190 A/common teal/Mongolia/2273/2011(H6N6)
KT327401 A/thrush/Taiwan/A234/2015(H5N2)
KC992218 A/ruddy shelduck/Mongolia/1787/2011(H3N8)
KC992237 A/common teal/Mongolia/2299/2011(H4N6)
LC339585 A/duck/Hokkaido/95/2014(H8N4)
MG976690 A/northern pintail/Alaska/UGAI16-3997/2016(H8N4)***
KC609865 A/wild duck/Korea/CSM42-34/2011(H7N9)
KC609860 A/wild duck/Korea/MHC35-25/2011(H7N9)
KC992199 A/common teal/Mongolia/1906/2011(H3N8)
JX534563 A/wild duck/Shandong/628/2011(H5N1)
KF260513 A/wild waterfowl/Hong Kong/MPP1311/2013(H2N9)
KF609525 A/tree sparrow/Shanghai/01/2013(H7N9)
KJ726730 A/wild chicken/Shanghai/C1/2014(H9N2)
KM076702 A/Anser fabalis/China/HuBS428/2014(H9N2)
JQ701710 A/marabou stork/Cambodia/TM068/2011(H5N1)
AB610970 A/whooper swan/Hokkaido/4/2011(H5N1)
KM251522 A/Anas crecca/Hubei/Chenhu1623-5/2014(H5N6)
KP674453 A/crow/India/01CA02/2014(H5N1)
CY235112 A/cinnamon teal/Idaho/UGAI16-1864/2016(H3N8)
CY166635 A/American green-winged teal/Wisconsin/11OS3425/2011(H12N5)
CY177067 A/green-winged teal/California/514/2013(H11N9)
MG280108 A/mallard duck/Arkansas/16OS3645/2016(H10N7)
MG599666 A/American black duck/Alberta/284/2016(H3N8)
CY134139 A/mallard/California/2566P/2011(H4N8)
KY550926 A/mallard/Kentucky/AH0051787/2016(H7N3)
MG280212 A/mallard duck/Ohio/16OS0869/2016(H7N3)
CY157316 A/mallard/California/3100/2012(H4N6)
CY177399 A/mallard/California/1495/2013(H5N5)
CY166425 A/mallard/Wisconsin/11OS4238/2011(H3N2)
CY166782 A/northern shoveler/Mississippi/11OS5875/2011(H5N2)
CY134373 A/northern pintail/California/183/2012(H1N1)
CY185951 A/blue-winged teal/Louisiana/AI11-2911/2011(H7N3)
CY132009 A/mallard/Ohio/11OS2045/2011(mixed)
CY157540 A/mallard/California/3370/2012(H9N2)
CY166272 A/mallard/Wisconsin/11OS4115/2011(H11N9)
CY167149 A/northern shoveler/Arkansas/12OS158/2012(H7N1)
CY133907 A/northern shoveler/California/2810/2011(H11N2)
CY149674 A/blue-winged teal/Canada/3237/2011(H4N6)
CY167084 A/long-tailed duck/Wisconsin/11OS4567/2011(H6N1)
MG279790 A/blue-winged teal/Ohio/16OS1027/2016(H3N6)
CY132571 A/mallard/Ohio/11OS1961/2011(H5N2)
CY166977 A/blue-winged teal/Missouri/11OS2640/2011(H4N8)
CY186845 A/blue-winged teal/Ohio/12OS2108/2012(mixed)
0.02

C

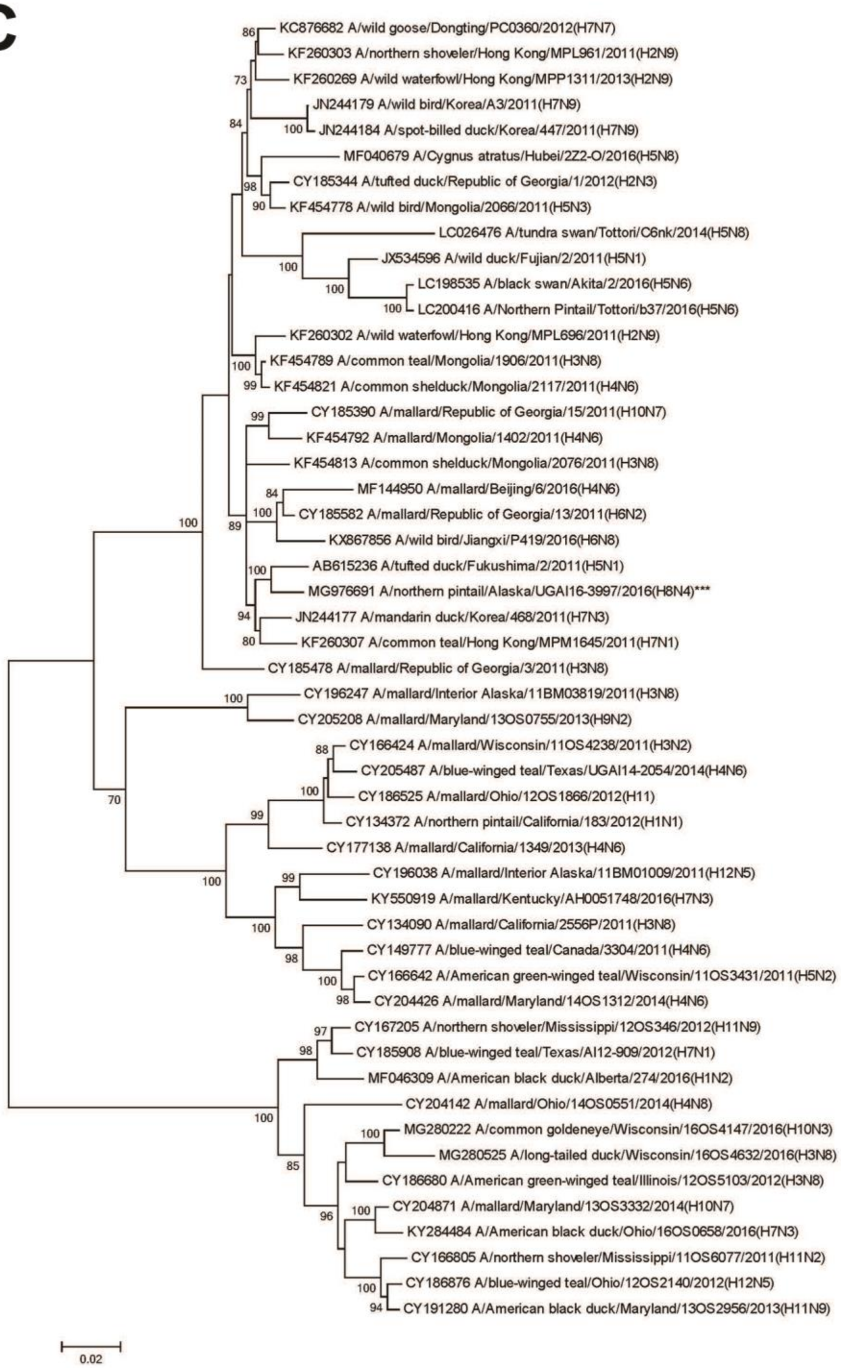
KC876682 A/wild goose/Dongting/PC0360/2012(H7N7)
KF260303 A/northern shoveler/Hong Kong/MPL961/2011(H2N9)
KF260269 A/wild waterfowl/Hong Kong/MPP1311/2013(H2N9)
JN244179 A/wild bird/Korea/A3/2011(H7N9)
JN244184 A/spot-billed duck/Korea/447/2011(H7N9)
MF040679 A/Cygnus atratus/Hubei/2Z2-O/2016(H5N8)
CY185344 A/tufted duck/Republic of Georgia/1/2012(H2N3)
KF454778 A/wild bird/Mongolia/2066/2011(H5N3)
LC026476 A/tundra swan/Tottori/C6nk/2014(H5N8)
JX534596 A/wild duck/Fujian/2/2011(H5N1)
LC198535 A/black swan/Akita/2/2016(H5N6)
LC200416 A/Northern Pintail/Tottori/b37/2016(H5N6)
KF260302 A/wild waterfowl/Hong Kong/MPL696/2011(H2N9)
KF454789 A/common teal/Mongolia/1906/2011(H3N8)
KF454821 A/common shelduck/Mongolia/2117/2011(H4N6)
CY185390 A/mallard/Republic of Georgia/15/2011(H10N7)
KF454792 A/mallard/Mongolia/1402/2011(H4N6)
KF454813 A/common shelduck/Mongolia/2076/2011(H3N8)
MF144950 A/mallard/Beijing/6/2016(H4N6)
CY185582 A/mallard/Republic of Georgia/13/2011(H6N2)
KX867856 A/wild bird/Jiangxi/P419/2016(H6N8)
AB615236 A/tufted duck/Fukushima/2/2011(H5N1)
MG976691 A/northern pintail/Alaska/UGAI16-3997/2016(H8N4)***
JN244177 A/mandarin duck/Korea/468/2011(H7N3)
KF260307 A/common teal/Hong Kong/MPM1645/2011(H7N1)
CY185478 A/mallard/Republic of Georgia/3/2011(H3N8)
CY196247 A/mallard/Interior Alaska/11BM03819/2011(H3N8)
CY205208 A/mallard/Maryland/13OS0755/2013(H9N2)
CY166424 A/mallard/Wisconsin/11OS4238/2011(H3N2)
CY205487 A/blue-winged teal/Texas/UGAI14-2054/2014(H4N6)
CY186525 A/mallard/Ohio/12OS1866/2012(H11)
CY134372 A/northern pintail/California/183/2012(H1N1)
CY177138 A/mallard/California/1349/2013(H4N6)
CY196038 A/mallard/Interior Alaska/11BM01009/2011(H12N5)
KY550919 A/mallard/Kentucky/AH0051748/2016(H7N3)
CY134090 A/mallard/California/2556P/2011(H3N8)
CY149777 A/blue-winged teal/Canada/3304/2011(H4N6)
CY166642 A/American green-winged teal/Wisconsin/11OS3431/2011(H5N2)
CY204426 A/mallard/Maryland/14OS1312/2014(H4N6)
CY167205 A/northern shoveler/Mississippi/12OS346/2012(H11N9)
CY185908 A/blue-winged teal/Texas/AI12-909/2012(H7N1)
MF046309 A/American black duck/Alberta/274/2016(H1N2)
CY204142 A/mallard/Ohio/14OS0551/2014(H4N8)
MG280222 A/common goldeneye/Wisconsin/16OS4147/2016(H10N3)
MG280525 A/long-tailed duck/Wisconsin/16OS4632/2016(H3N8)
CY186680 A/American green-winged teal/Illinois/12OS5103/2012(H3N8)
CY204871 A/mallard/Maryland/13OS3332/2014(H10N7)
KY284484 A/American black duck/Ohio/16OS0658/2016(H7N3)
CY166805 A/northern shoveler/Mississippi/11OS6077/2011(H11N2)
CY186876 A/blue-winged teal/Ohio/12OS2140/2012(H12N5)
CY191280 A/American black duck/Maryland/13OS2956/2013(H11N9)
0.02

D

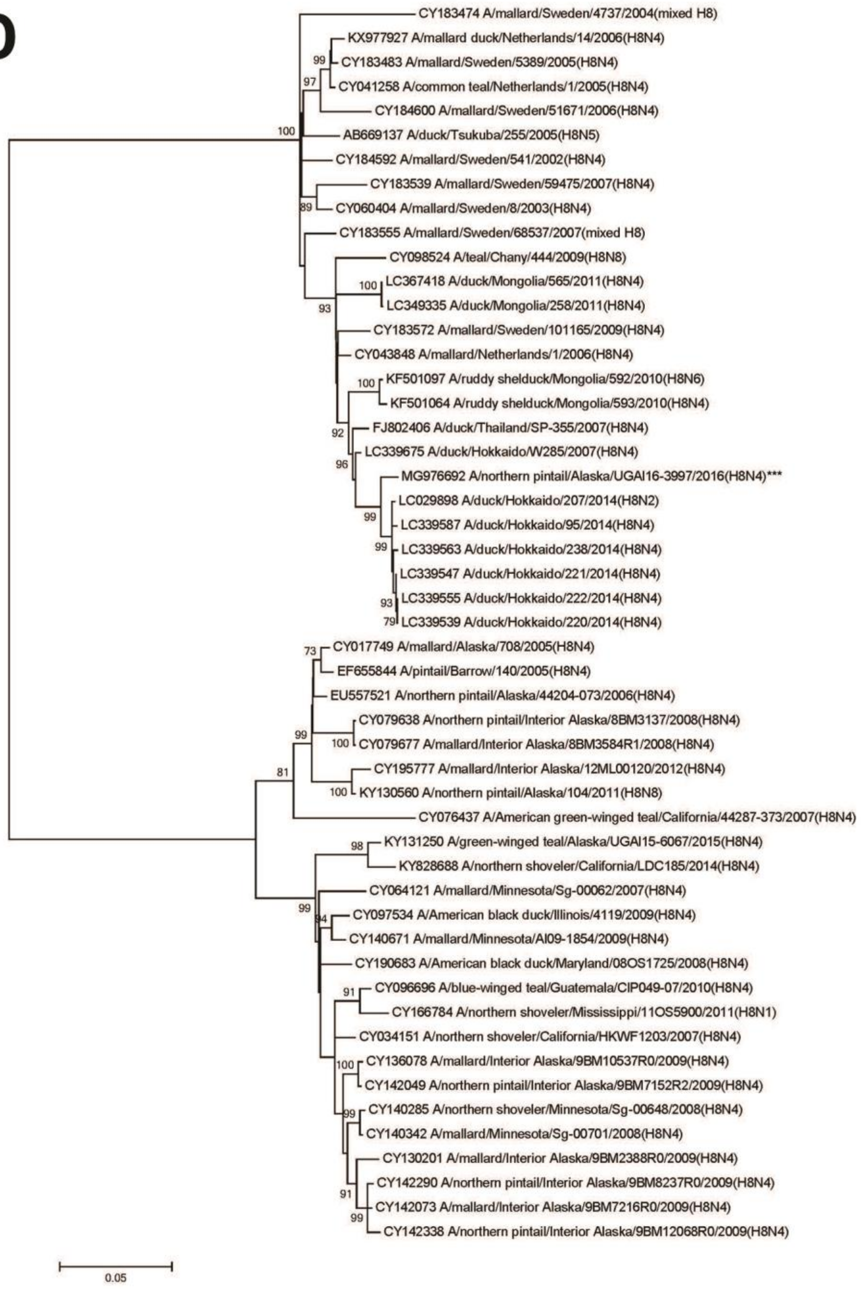

CY183474 A/mallard/Sweden/4737/2004(mixed H8)
KX977927 A/mallard duck/Netherlands/14/2006(H8N4)
CY183483 A/mallard/Sweden/5389/2005(H8N4)
CY041258 A/common teal/Netherlands/1/2005(H8N4)
CY184600 A/mallard/Sweden/51671/2006(H8N4)
AB669137 A/duck/Tsukuba/255/2005(H8N5)
CY184592 A/mallard/Sweden/541/2002(H8N4)
CY183539 A/mallard/Sweden/59475/2007(H8N4)
CY060404 A/mallard/Sweden/8/2003(H8N4)
CY183555 A/mallard/Sweden/68537/2007(mixed H8)
CY098524 A/teal/Chany/444/2009(H8N8)
LC367418 A/duck/Mongolia/565/2011(H8N4)
LC349335 A/duck/Mongolia/258/2011(H8N4)
CY183572 A/mallard/Sweden/101165/2009(H8N4)
CY043848 A/mallard/Netherlands/1/2006(H8N4)
KF501097 A/ruddy shelduck/Mongolia/592/2010(H8N6)
KF501064 A/ruddy shelduck/Mongolia/593/2010(H8N4)
FJ802406 A/duck/Thailand/SP-355/2007(H8N4)
LC339675 A/duck/Hokkaido/W285/2007(H8N4)
MG976692 A/northern pintail/Alaska/UGAI16-3997/2016(H8N4)***
LC029898 A/duck/Hokkaido/207/2014(H8N2)
LC339587 A/duck/Hokkaido/95/2014(H8N4)
LC339563 A/duck/Hokkaido/238/2014(H8N4)
LC339547 A/duck/Hokkaido/221/2014(H8N4)
LC339555 A/duck/Hokkaido/222/2014(H8N4)
LC339539 A/duck/Hokkaido/220/2014(H8N4)
CY017749 A/mallard/Alaska/708/2005(H8N4)
EF655844 A/pintail/Barrow/140/2005(H8N4)
EU557521 A/northern pintail/Alaska/44204-073/2006(H8N4)
CY079638 A/northern pintail/Interior Alaska/8BM3137/2008(H8N4)
CY079677 A/mallard/Interior Alaska/8BM3584R1/2008(H8N4)
CY195777 A/mallard/Interior Alaska/12ML00120/2012(H8N4)
KY130560 A/northern pintail/Alaska/104/2011(H8N8)
CY076437 A/American green-winged teal/California/44287-373/2007(H8N4)
KY131250 A/green-winged teal/Alaska/UGAI15-6067/2015(H8N4)
KY828688 A/northern shoveler/California/LDC185/2014(H8N4)
CY064121 A/mallard/Minnesota/Sg-00062/2007(H8N4)
CY097534 A/American black duck/Illinois/4119/2009(H8N4)
CY140671 A/mallard/Minnesota/AI09-1854/2009(H8N4)
CY190683 A/American black duck/Maryland/08OS1725/2008(H8N4)
CY096696 A/blue-winged teal/Guatemala/CIP049-07/2010(H8N4)
CY166784 A/northern shoveler/Mississippi/11OS5900/2011(H8N1)
CY034151 A/northern shoveler/California/HKWF1203/2007(H8N4)
CY136078 A/mallard/Interior Alaska/9BM10537R0/2009(H8N4)
CY142049 A/northern pintail/Interior Alaska/9BM7152R2/2009(H8N4)
CY140285 A/northern shoveler/Minnesota/Sg-00648/2008(H8N4)
CY140342 A/mallard/Minnesota/Sg-00701/2008(H8N4)
CY130201 A/mallard/Interior Alaska/9BM2388R0/2009(H8N4)
CY142290 A/northern pintail/Interior Alaska/9BM8237R0/2009(H8N4)
CY142073 A/mallard/Interior Alaska/9BM7216R0/2009(H8N4)
CY142338 A/northern pintail/Interior Alaska/9BM12068R0/2009(H8N4)
0.05

E

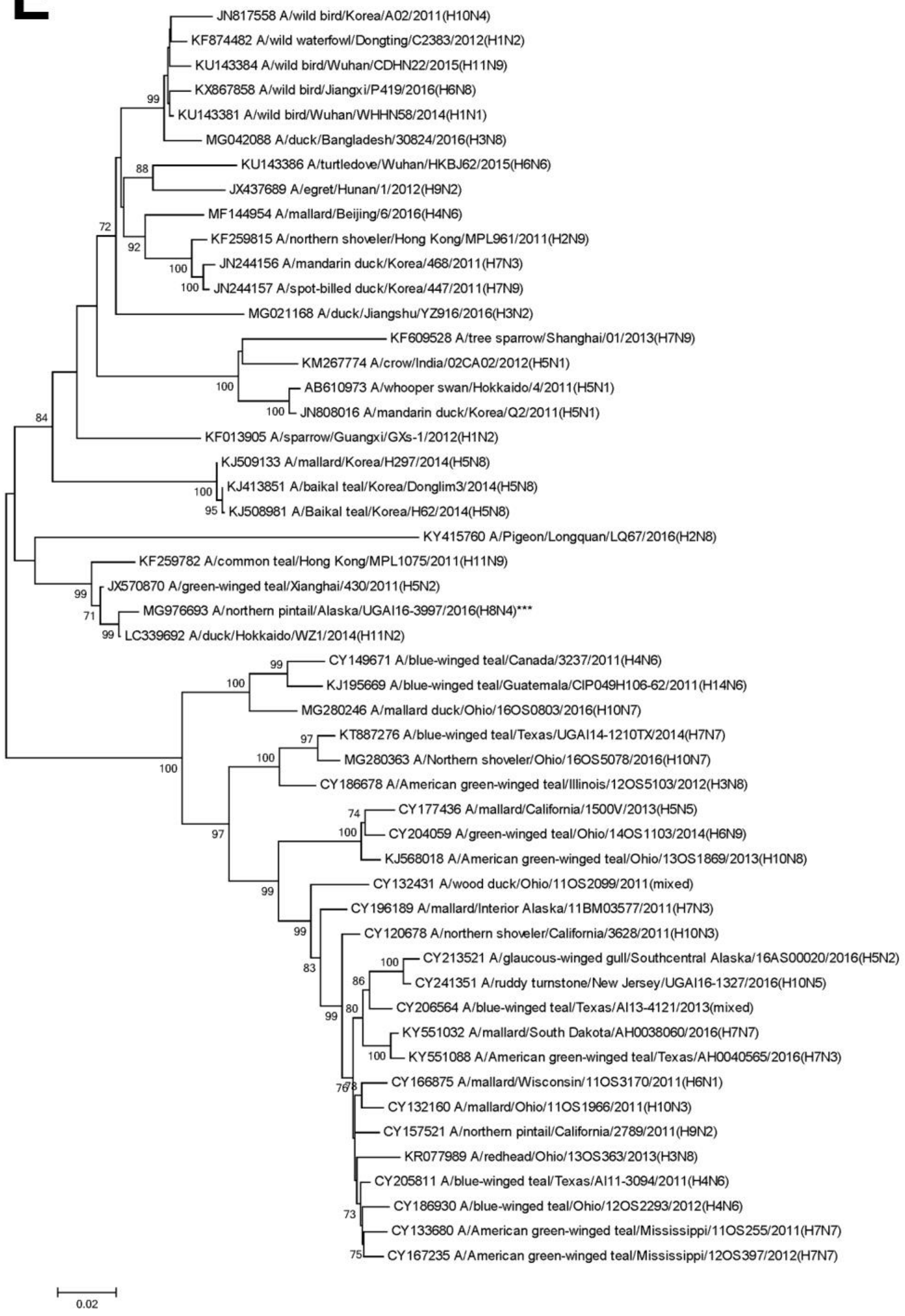
JN817558 A/wild bird/Korea/A02/2011(H10N4)
KF874482 A/wild waterfowl/Dongting/C2383/2012(H1N2)
KU143384 A/wild bird/Wuhan/CDHN22/2015(H11N9)
KX867858 A/wild bird/Jiangxi/P419/2016(H6N8)
KU143381 A/wild bird/Wuhan/WHHN58/2014(H1N1)
MG042088 A/duck/Bangladesh/30824/2016(H3N8)
KU143386 A/turtledove/Wuhan/HKBJ62/2015(H6N6)
JX437689 A/egret/Hunan/1/2012(H9N2)
MF144954 A/mallard/Beijing/6/2016(H4N6)
KF259815 A/northern shoveler/Hong Kong/MPL961/2011(H2N9)
JN244156 A/mandarin duck/Korea/468/2011(H7N3)
JN244157 A/spot-billed duck/Korea/447/2011(H7N9)
MG021168 A/duck/Jiangshu/YZ916/2016(H3N2)
KF609528 A/tree sparrow/Shanghai/01/2013(H7N9)
KM267774 A/crow/India/02CA02/2012(H5N1)
AB610973 A/whooper swan/Hokkaido/4/2011(H5N1)
JN808016 A/mandarin duck/Korea/Q2/2011(H5N1)
KF013905 A/sparrow/Guangxi/GXs-1/2012(H1N2)
KJ509133 A/mallard/Korea/H297/2014(H5N8)
KJ413851 A/baikal teal/Korea/Donglim3/2014(H5N8)
KJ508981 A/Baikal teal/Korea/H62/2014(H5N8)
KY415760 A/Pigeon/Longquan/LQ67/2016(H2N8)
KF259782 A/common teal/Hong Kong/MPL1075/2011(H11N9)
JX570870 A/green-winged teal/Xianghai/430/2011(H5N2)
MG976693 A/northern pintail/Alaska/UGAI16-3997/2016(H8N4)***
LC339692 A/duck/Hokkaido/WZ1/2014(H11N2)
CY149671 A/blue-winged teal/Canada/3237/2011(H4N6)
KJ195669 A/blue-winged teal/Guatemala/CIP049H106-62/2011(H14N6)
MG280246 A/mallard duck/Ohio/16OS0803/2016(H10N7)
KT887276 A/blue-winged teal/Texas/UGAI14-1210TX/2014(H7N7)
MG280363 A/Northern shoveler/Ohio/16OS5078/2016(H10N7)
CY186678 A/American green-winged teal/Illinois/12OS5103/2012(H3N8)
CY177436 A/mallard/California/1500V/2013(H5N5)
CY204059 A/green-winged teal/Ohio/14OS1103/2014(H6N9)
KJ568018 A/American green-winged teal/Ohio/13OS1869/2013(H10N8)
CY132431 A/wood duck/Ohio/11OS2099/2011(mixed)
CY196189 A/mallard/Interior Alaska/11BM03577/2011(H7N3)
CY120678 A/northern shoveler/California/3628/2011(H10N3)
CY213521 A/glaucous-winged gull/Southcentral Alaska/16AS00020/2016(H5N2)
CY241351 A/ruddy turnstone/New Jersey/UGAI16-1327/2016(H10N5)
CY206564 A/blue-winged teal/Texas/AI13-4121/2013(mixed)
KY551032 A/mallard/South Dakota/AH0038060/2016(H7N7)
KY551088 A/American green-winged teal/Texas/AH0040565/2016(H7N3)
CY166875 A/mallard/Wisconsin/11OS3170/2011(H6N1)
CY132160 A/mallard/Ohio/11OS1966/2011(H10N3)
CY157521 A/northern pintail/California/2789/2011(H9N2)
KR077989 A/redhead/Ohio/13OS363/2013(H3N8)
CY205811 A/blue-winged teal/Texas/AI11-3094/2011(H4N6)
CY186930 A/blue-winged teal/Ohio/12OS2293/2012(H4N6)
CY133680 A/American green-winged teal/Mississippi/11OS255/2011(H7N7)
CY167235 A/American green-winged teal/Mississippi/12OS397/2012(H7N7)
0.02

F

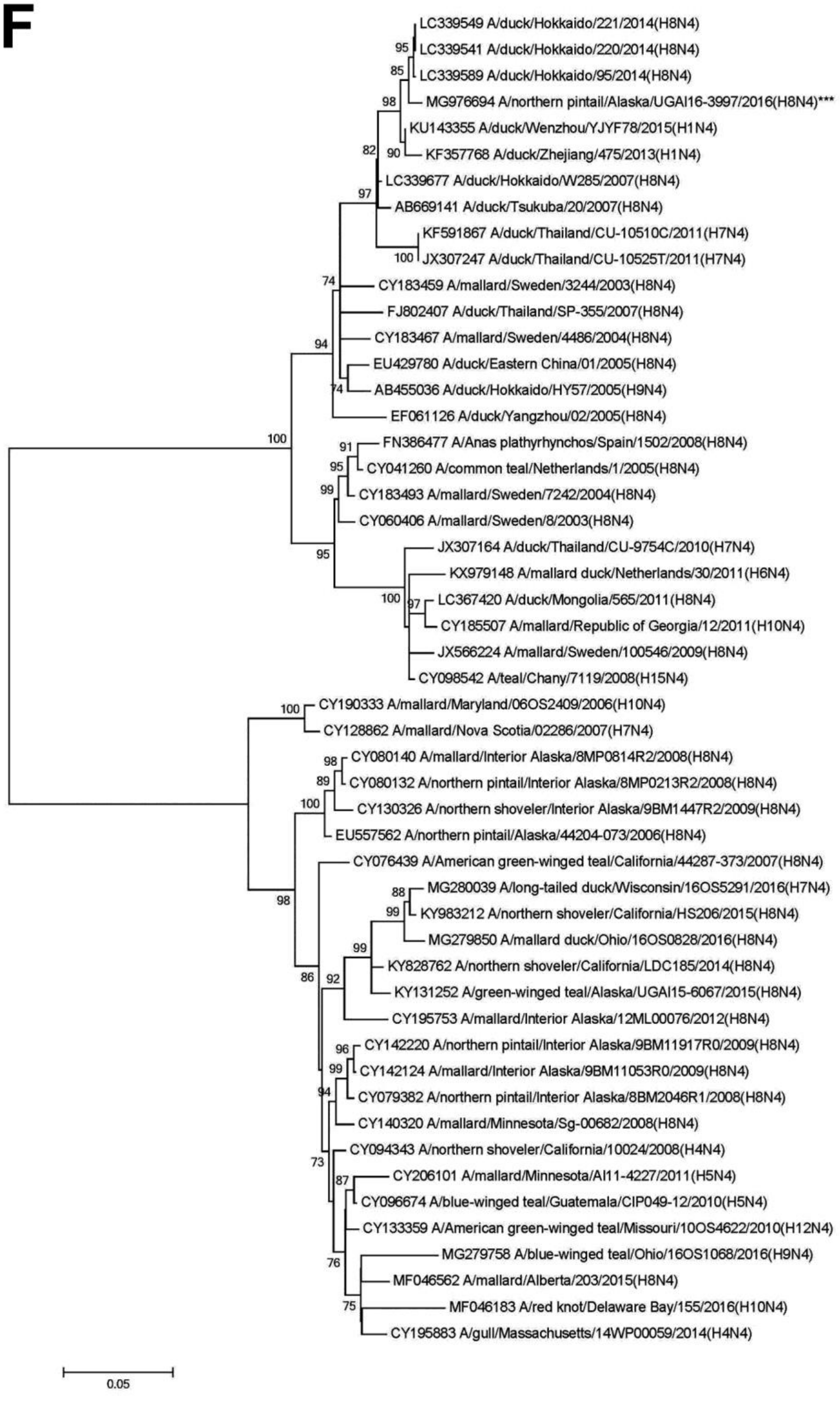

LC339549 A/duck/Hokkaido/221/2014(H8N4)
LC339541 A/duck/Hokkaido/220/2014(H8N4)
LC339589 A/duck/Hokkaido/95/2014(H8N4)
MG976694 A/northern pintail/Alaska/UGAI16-3997/2016(H8N4)***
KU143355 A/duck/Wenzhou/YJYF78/2015(H1N4)
KF357768 A/duck/Zhejiang/475/2013(H1N4)
LC339677 A/duck/Hokkaido/W285/2007(H8N4)
AB669141 A/duck/Tsukuba/20/2007(H8N4)
KF591867 A/duck/Thailand/CU-10510C/2011(H7N4)
JX307247 A/duck/Thailand/CU-10525T/2011(H7N4)
CY183459 A/mallard/Sweden/3244/2003(H8N4)
FJ802407 A/duck/Thailand/SP-355/2007(H8N4)
CY183467 A/mallard/Sweden/4486/2004(H8N4)
EU429780 A/duck/Eastern China/01/2005(H8N4)
AB455036 A/duck/Hokkaido/HY57/2005(H9N4)
EF061126 A/duck/Yangzhou/02/2005(H8N4)
FN386477 A/Anas plathyrhynchos/Spain/1502/2008(H8N4)
CY041260 A/common teal/Netherlands/1/2005(H8N4)
CY183493 A/mallard/Sweden/7242/2004(H8N4)
CY060406 A/mallard/Sweden/8/2003(H8N4)
JX307164 A/duck/Thailand/CU-9754C/2010(H7N4)
KX979148 A/mallard duck/Netherlands/30/2011(H6N4)
LC367420 A/duck/Mongolia/565/2011(H8N4)
CY185507 A/mallard/Republic of Georgia/12/2011(H10N4)
JX566224 A/mallard/Sweden/100546/2009(H8N4)
CY098542 A/teal/Chany/7119/2008(H15N4)
CY190333 A/mallard/Maryland/06OS2409/2006(H10N4)
CY128862 A/mallard/Nova Scotia/02286/2007(H7N4)
CY080140 A/mallard/Interior Alaska/8MP0814R2/2008(H8N4)
CY080132 A/northern pintail/Interior Alaska/8MP0213R2/2008(H8N4)
CY130326 A/northern shoveler/Interior Alaska/9BM1447R2/2009(H8N4)
EU557562 A/northern pintail/Alaska/44204-073/2006(H8N4)
CY076439 A/American green-winged teal/California/44287-373/2007(H8N4)
MG280039 A/long-tailed duck/Wisconsin/16OS5291/2016(H7N4)
KY983212 A/northern shoveler/California/HS206/2015(H8N4)
MG279850 A/mallard duck/Ohio/16OS0828/2016(H8N4)
KY828762 A/northern shoveler/California/LDC185/2014(H8N4)
KY131252 A/green-winged teal/Alaska/UGAI15-6067/2015(H8N4)
CY195753 A/mallard/Interior Alaska/12ML00076/2012(H8N4)
CY142220 A/northern pintail/Interior Alaska/9BM11917R0/2009(H8N4)
CY142124 A/mallard/Interior Alaska/9BM11053R0/2009(H8N4)
CY079382 A/northern pintail/Interior Alaska/8BM2046R1/2008(H8N4)
CY140320 A/mallard/Minnesota/Sg-00682/2008(H8N4)
CY094343 A/northern shoveler/California/10024/2008(H4N4)
CY206101 A/mallard/Minnesota/AI11-4227/2011(H5N4)
CY096674 A/blue-winged teal/Guatemala/CIP049-12/2010(H5N4)
CY133359 A/American green-winged teal/Missouri/10OS4622/2010(H12N4)
MG279758 A/blue-winged teal/Ohio/16OS1068/2016(H9N4)
MF046562 A/mallard/Alberta/203/2015(H8N4)
MF046183 A/red knot/Delaware Bay/155/2016(H10N4)
CY195883 A/gull/Massachusetts/14WP00059/2014(H4N4)
0.05

G

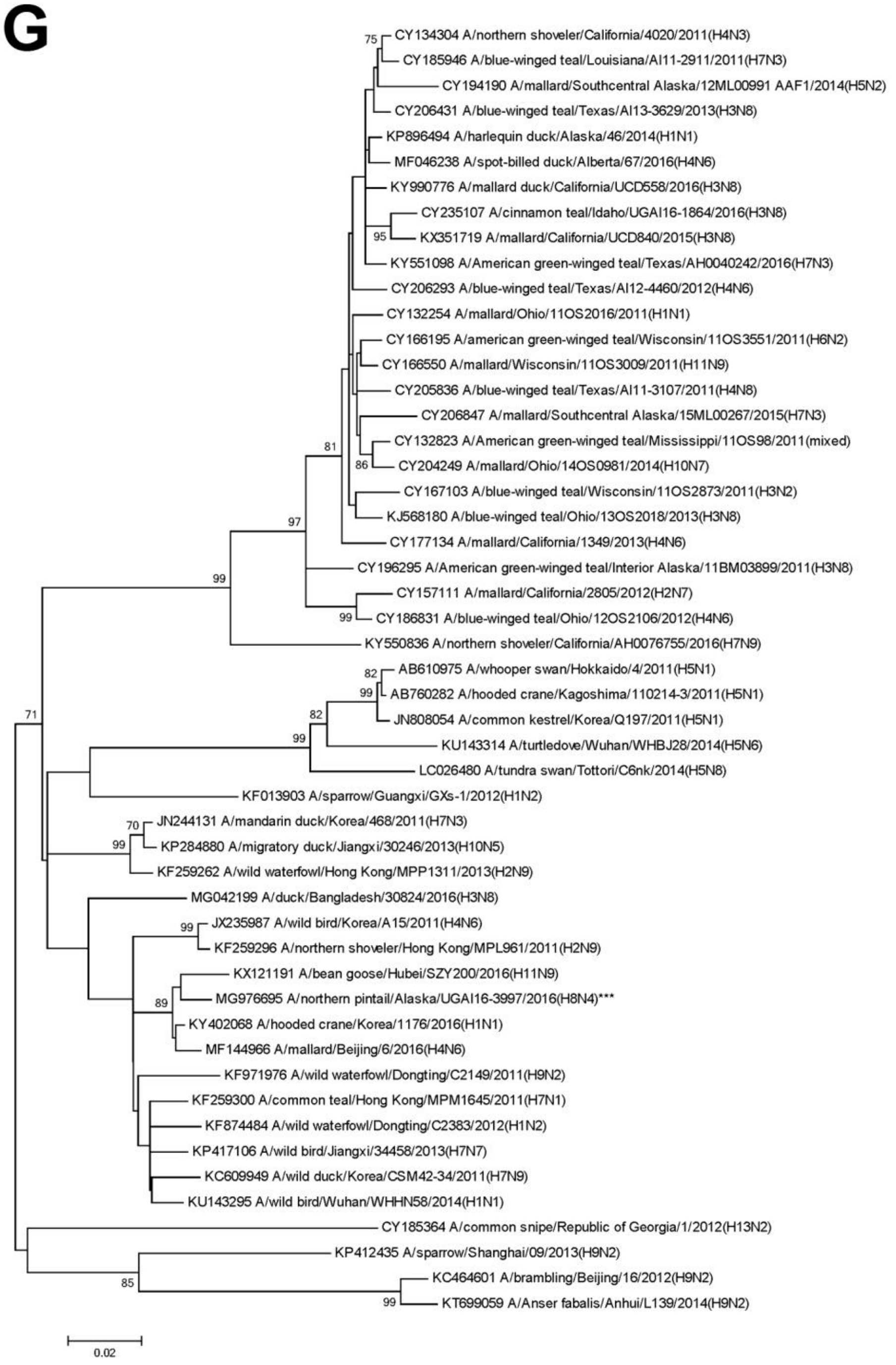

CY134304 A/northern shoveler/California/4020/2011(H4N3)
CY185946 A/blue-winged teal/Louisiana/AI11-2911/2011(H7N3)
CY194190 A/mallard/Southcentral Alaska/12ML00991 AAF1/2014(H5N2)
CY206431 A/blue-winged teal/Texas/AI13-3629/2013(H3N8)
KP896494 A/harlequin duck/Alaska/46/2014(H1N1)
MF046238 A/spot-billed duck/Alberta/67/2016(H4N6)
KY990776 A/mallard duck/California/UCD558/2016(H3N8)
CY235107 A/cinnamon teal/Idaho/UGAI16-1864/2016(H3N8)
KX351719 A/mallard/California/UCD840/2015(H3N8)
KY551098 A/American green-winged teal/Texas/AH0040242/2016(H7N3)
CY206293 A/blue-winged teal/Texas/AI12-4460/2012(H4N6)
CY132254 A/mallard/Ohio/11OS2016/2011(H1N1)
CY166195 A/american green-winged teal/Wisconsin/11OS3551/2011(H6N2)
CY166550 A/mallard/Wisconsin/11OS3009/2011(H11N9)
CY205836 A/blue-winged teal/Texas/AI11-3107/2011(H4N8)
CY206847 A/mallard/Southcentral Alaska/15ML00267/2015(H7N3)
CY132823 A/American green-winged teal/Mississippi/11OS98/2011(mixed)
CY204249 A/mallard/Ohio/14OS0981/2014(H10N7)
CY167103 A/blue-winged teal/Wisconsin/11OS2873/2011(H3N2)
KJ568180 A/blue-winged teal/Ohio/13OS2018/2013(H3N8)
CY177134 A/mallard/California/1349/2013(H4N6)
CY196295 A/American green-winged teal/Interior Alaska/11BM03899/2011(H3N8)
CY157111 A/mallard/California/2805/2012(H2N7)
CY186831 A/blue-winged teal/Ohio/12OS2106/2012(H4N6)
KY550836 A/northern shoveler/California/AH0076755/2016(H7N9)
AB610975 A/whooper swan/Hokkaido/4/2011(H5N1)
AB760282 A/hooded crane/Kagoshima/110214-3/2011(H5N1)
JN808054 A/common kestrel/Korea/Q197/2011(H5N1)
KU143314 A/turtledove/Wuhan/WHBJ28/2014(H5N6)
LC026480 A/tundra swan/Tottori/C6nk/2014(H5N8)
KF013903 A/sparrow/Guangxi/GXs-1/2012(H1N2)
JN244131 A/mandarin duck/Korea/468/2011(H7N3)
KP284880 A/migratory duck/Jiangxi/30246/2013(H10N5)
KF259262 A/wild waterfowl/Hong Kong/MPP1311/2013(H2N9)
MG042199 A/duck/Bangladesh/30824/2016(H3N8)
JX235987 A/wild bird/Korea/A15/2011(H4N6)
KF259296 A/northern shoveler/Hong Kong/MPL961/2011(H2N9)
KX121191 A/bean goose/Hubei/SZY200/2016(H11N9)
MG976695 A/northern pintail/Alaska/UGAI16-3997/2016(H8N4)***
KY402068 A/hooded crane/Korea/1176/2016(H1N1)
MF144966 A/mallard/Beijing/6/2016(H4N6)
KF971976 A/wild waterfowl/Dongting/C2149/2011(H9N2)
KF259300 A/common teal/Hong Kong/MPM1645/2011(H7N1)
KF874484 A/wild waterfowl/Dongting/C2383/2012(H1N2)
KP417106 A/wild bird/Jiangxi/34458/2013(H7N7)
KC609949 A/wild duck/Korea/CSM42-34/2011(H7N9)
KU143295 A/wild bird/Wuhan/WHHN58/2014(H1N1)
CY185364 A/common snipe/Republic of Georgia/1/2012(H13N2)
KP412435 A/sparrow/Shanghai/09/2013(H9N2)
KC464601 A/brambling/Beijing/16/2012(H9N2)
KT699059 A/Anser fabalis/Anhui/L139/2014(H9N2)
0.02

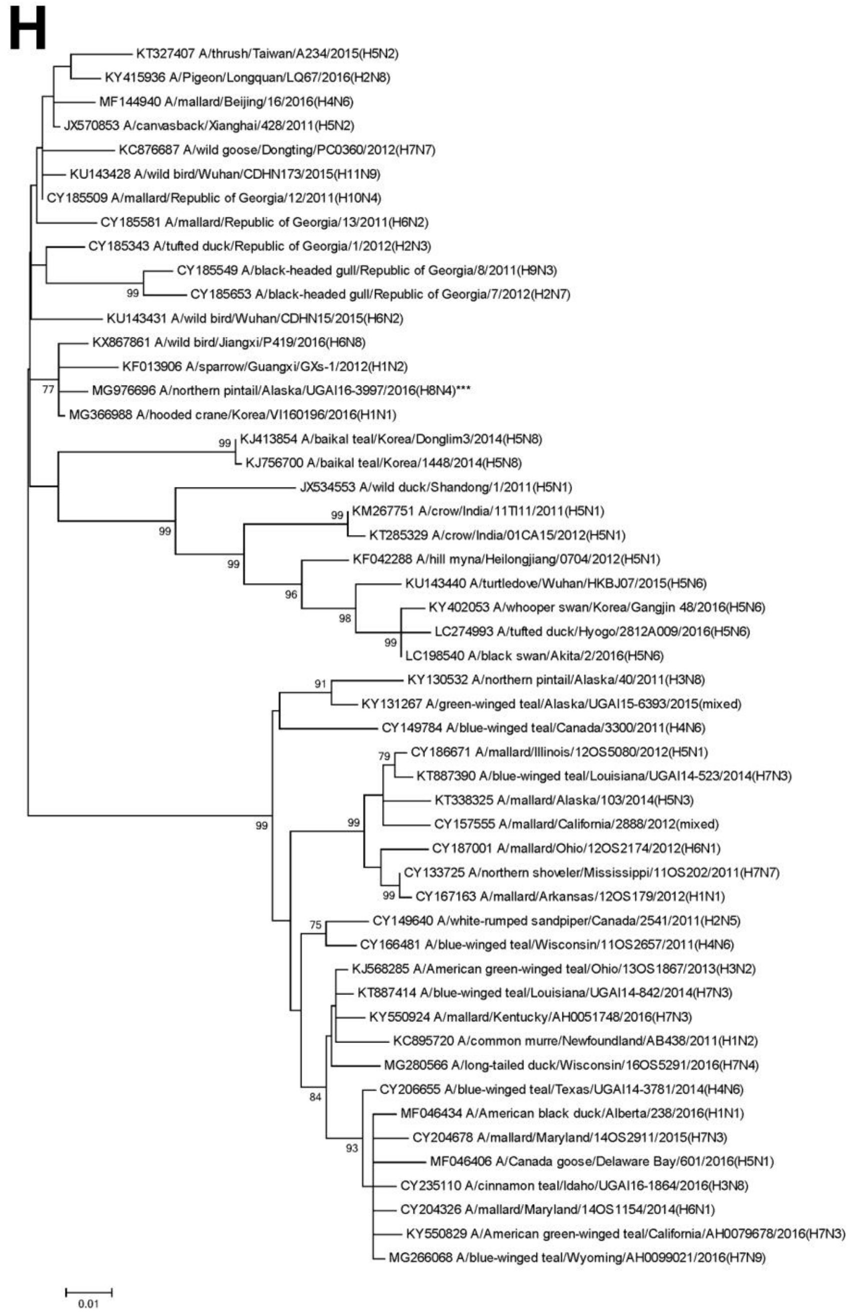

Technical Appendix Figure. Unrooted maximum-likelihood phylogenetic trees with complete strain names as tip labels showing inferred relationship among nucleotide sequences for the complete coding regions of the gene segments for influenza A virus strain A/northern pintail/Alaska/UGAI16–3997/2016(H8N4) (indicated with 3 asterisks) and reference sequences from viruses isolated from birds in Eurasia and North America. Bootstrap support values $\geq$70 are shown. H, hemagglutinin; M, matrix; N, neuraminidase; NP, nucleoprotein; NS, nonstructural; PA, polymerase acidic; PB, polymerase basic. Scale bar indicates nucleotide substitutions per site.
